# Editorial: Biostimulants in Agriculture

**DOI:** 10.3389/fpls.2020.00040

**Published:** 2020-02-04

**Authors:** Youssef Rouphael, Giuseppe Colla

**Affiliations:** ^1^ Department of Agricultural Sciences, University of Naples Federico II, Portici, Italy; ^2^ Department of Agriculture and Forest Sciences, University of Tuscia, Viterbo, Italy

**Keywords:** humic acids, mycorrhizal fungi, nutrient use efficiency, physiological and molecular mechanisms, high-throughput phenotyping, plant growth promoting rhizobacteria, protein hydrolysates, seaweed extracts

## Plant Biostimulants: Rationale, State of the Art and Evolution

Recently, the agricultural sector is facing concomitant challenges of rising the productivity to feed the growing global population and increasing the resources use efficiency, while reducing the environmental impact on the ecosystems and human health. In fact, fertilizers and pesticides play a crucial role in agriculture, representing a powerful tool for growers to increase yield and guarantee continuous productivity throughout the seasons under both optimal and suboptimal conditions. In the last three decades, several technological innovations have been proposed to enhance the sustainability of agricultural production systems, through a significant reduction of synthetic agrochemicals like pesticides and fertilizers. A promising and environmental-friendly innovation would be the use of natural plant biostimulants (PBs) that enhance flowering, plant growth, fruit set, crop productivity, and nutrient use efficiency (NUE), and are able also to improve the tolerance against a wide range of abiotic stressors (Colla and Rouphael, 2015). PBs were initially defined by excluding some functionalities like fertilizers or plant protection products. In 1997, in Grounds Maintenance web-journal, Zhang and Schmidt from the Department of Crop and Soil Environmental Sciences of the Virginia Polytechnic Institute and State University, defined PBs as “materials that, in minute quantities, promote plant growth”. By using the statement “minute quantities” for describing PBs, the authors implicitly wanted to discriminate biostimulants from nutrients and soil amendments, which also promote plant growth, but are clearly applied in larger quantities. The PBs mentioned in this web article were two important categories such as humic acids and seaweed extracts, and their action on plants was proposed to be essentially hormonal. In 2012, the European Commission has assigned an *ad hoc* study on plant biostimulants to evaluate the substances and materials involved, which was published by du Jardin (2012) as: “The Science of Plant Biostimulants - A bibliographic Analysis”. Based on the scientific literature (250 scientific articles using the term ‘biostimulant' in their titles and/or abstracts), the following definition was proposed: “Plant biostimulants are substances and materials, with the exception of nutrients and pesticides, which, when applied to plant, seeds or growing substrates in specific formulations, have the capacity to modify physiological processes of plants in a way that provides potential benefits to growth, development and/or stress responses”. du Jardin (2012) concluded that PBs are very heterogeneous materials, and proposed in his study eight categories of substances that acts as biostimulants: humic substances, complex organic materials (obtained from agro-industrial and urban waste products, sewage sludge extracts, composts, and manure), beneficial chemical elements (Al, Co, Na, Se, and Si), inorganic salts including phosphite, seaweed extracts (brown, red, and green macroalgae), chitin and chitosan derivates, antitranspirants (kaolin and polyacrylamide), and free amino acids and N-containing substances (peptides, polyamines, and betaines); but did not include any microbial biostimulants. Three years later in the frame of a special issue on “Biostimulants in Horticulture” conducted by Colla and Rouphael (2015), a new definition was proposed by du Jardin (2015), which was supported by scientific evidence about the mode of action, nature and types of effects of PBs on agricultural and horticultural crops. PBs were defined by du Jardin (2015) as follows: “A plant biostimulant is any substance or microorganism applied to plants with the aim to enhance nutrition efficiency, abiotic stress tolerance and/or crop quality traits, regardless of its nutrient content”. This definition could be completed by “By extension plant biostimulants also designate commercial products containing mixtures of such substances and/or microorganisms”. In their special issue Colla and Rouphael (2015) proposed 6 non-microbial and 3 microbial categories of PBs: (i) chitosan (Pichyangkura and Chadchawan, 2015), (ii) humic and fulvic acids (Canellas et al., 2015), (iii) protein hydrolysates (Colla et al., 2015), (iv) phosphites (Gómez-Merino and Trejo-Téllez, 2015), (v) seaweed extracts (Battacharyya et al., 2015), (vi) silicon (Savvas and Ntatsi, 2015), (vii) arbuscular mycorrhizal fungi (AMF; Rouphael et al., 2015), (viii) plant growth-promoting rhizobacteria (PGPR; Ruzzi and Aroca, 2015), and (ix) *Trichoderma* spp. (López-Bucio et al., 2015).

The definition of PBs has been rigorously debated over the last decade, and recently under the new Regulation (EU) 2019/1009, which led to the following: “A plant biostimulant shall be an EU fertilising product the function of which is to stimulate plant nutrition processes independently of the product's nutrient content with the sole aim of improving one or more of the following characteristics of the plant or the plant rhizosphere: i) nutrient use efficiency, ii) tolerance to abiotic stress, iii) quality traits, or iv) availability of confined nutrients in the soil or rhizosphere” (EU, 2019). Based on this definition, PBs are specified on the basis of agricultural functions claims, and include diverse bioactive natural substances: (i) humic and fulvic acids, (ii) animal and vegetal protein hydrolysates, (iii) macroalgae seaweeds extracts, and (iv) silicon, as well as beneficial microorganisms: (i) arbuscular mycorrhizal fungi (AMF) and (ii) N-fixing bacteria of strains belonging to the genera *Rhizobium*, *Azotobacter*, and *Azospirillum*. However, the justification of agricultural claims of a given microbial or non-microbial biostimulant, is considered an important element to allow PBs to be placed on the European Union market; thus members of the European Biostimulant Industry Council (Ricci et al.) proposed general principles and guidelines for trials and assays to follow when justifying PBs claims, that were outlined in details in their policy and practice review article.

More than 700 scientific papers were published in the last 10 years (2009–2019) on “plant biostimulants” (www.scopus.com), where several researchers were able to demonstrate that microbial and non-microbial PBs are capable of inducing an array of morpho-anatomical, biochemical, physiological, and molecular plant responses such as boosting crop productivity, NUE, and increasing tolerance against abiotic stresses (Calvo et al., 2014; Haplern et al., 2015; Nardi et al., 2016; De Pascale et al., 2017; Rouphael et al., 2017a; Rouphael et al., 2017b; Rouphael et al., 2017c; Yakhin et al., 2017; Rouphael et al., 2018a).

This Research Topic collected 50 scientific contributions from high qualified research groups working on PBs and covering the molecular, cellular, and physiological mechanisms underlying plant-biostimulant interactions under different environment and management strategies. Moreover, the present Research Topic compiles many aspects that are helpful to the scientific community, extension specialists, and commercial enterprises to better elucidate the causal/functional mechanism of microbial and non-microbial biostimulants. The elucidation of the agricultural function (i.e., improve nutrient use efficiency, quality, and tolerance to abiotic stresses) and action mechanisms of PBs will permit to develop a second generation of biostimulants where synergies and complementary mechanism can be functionally designed.

## Non-Microbial and Microbial Plant Biostimulants

Protein hydrolysates (PHs) which contain mainly signaling peptides and free amino acids have gained prominence as non-microbial PBs because of their potential to enhance germination, seedling growth, plant growth, fruits, and vegetables quality as well as crop productivity especially under environmental stress conditions (Colla et al.). In their review paper, the authors aimed at uncovering the physiological and molecular mechanisms behind the biostimulant action of animal or vegetal-based PHs on a wide range of agricultural and horticultural crops. Interestingly, the authors also provided for the first-time evidence that plant microbiomes are modified by the application of PHs, and some of the benefits derived from these products might be due in part to changes in the quanti-qualitative composition and activity of these microbial communities.

Seaweed extracts (SWE) represent another important category of organic non-microbial PBs; however red, green, and brown macroalgae are the most common SWE used in agriculture and horticulture with several commercial products present on the market. Macroalgae are typically harvested from seas and oceans, which hampers the chemical composition and quality of its raw material, leading to difficulties in standardization and getting reliable performance of the extracted products. Therefore, searching for controlled production of *in-house algae* is an urgent need for the scientific community and private companies. Chiaiese et al. proposed microalgae as a renewable source of PBs. In their review paper, the authors described the extraction techniques and the bioactive compounds (carbohydrates, proteins, and amino acids) as well as the biostimulatory action of microalgal extracts belonging to the following species: *Chlorella vulgaris*, *Acutodesmus dimorphus*, *S. platensis*, *Scenedesmus quadricauda*, *Dunaliella salina*, *Chlorella ellipsoida*, *Spirulina maxima*, and *Calothrix elenkinii*. On the other hand, developing PBs from agro-food and industrial by-products could also open new opportunities in a full circular economy approach. Xu and Geelen reviewed examples of PBs derived from agricultural by-products and identified the important criteria to select potential by-products for developing efficient PBs. These criteria included: absence of pesticides and heavy metals, collection and storage at low cost and sufficient availability all year round. Several examples of PBs derived from agricultural and industrial by-products including vermicompost, composted urban waste, sewage sludge, PH, and chitin/chitosan derivatives were discussed in detail.

In addition to non-microbial PBs, the use of microbial PBs such as PGPR and AMF are highly considered as sustainable and efficient tools for securing yield stability under low-input conditions in particular N and P deficiency (i.e., biofertilizer effects), but also as a innovative technology to improve crop tolerance to abiotic stressors in particular extreme temperatures, drought and salinity. In their review papers Backer et al., Granada et al. and Bitterlich et al. described the mechanisms of these beneficial microorganisms regarding nutrient uptake (especially N and P) and tolerance to environmental stress including signals exchange between plant roots and PGPR and AMF. Particularly, Granada et al. reported that the reduction of P-fertilization could be achieved with the use of high efficient P-solubilizing bacterial isolates as crop inoculants. Moreover, based on a long-term study (7 years), Lu et al. reported that no-tillage with straw return had a protective effect on AMF community structures compared to conventional moldboard-plowing or tillage without straw, thereby playing a crucial role in the development of agricultural sustainability in China. In line with Backer et al. and Bitterlich et al. reviews, Turrini et al. elucidated the functional complementarity of AMF and associated microbiota. Particularly, the authors revealed the functional roles of plant growth promoting bacteria (PGPB) and mycorrhizal helper bacteria (MHB), that promote AMF activity and development and thus boost crop productivity under both optimal and sub-optimal conditions. Similarly, Agnolucci et al. demonstrated by using a polyphasic approach (a combination of culture-dependent analyses and metagenomic sequencing.), that AMF inoculum (*Rhizoglomus irregulare* BEG72) is home of a large and diverse community of bacteria with important functional PGP traits (i.e., solubilizing phosphate and producing siderophores and indole acetic acid), and possibly acting in synergy with AMF and providing beneficial effects on crop performance. Finally, Woo and Pepe reported that designing and developing potential *agricultural probiotics* such as *Trichoderma*-*Azotobacter* consortia is a priority for the PBs sector and should be adopted as a sustainable crop management strategy to improve yield and its qualitative aspect.

## Implications of Biostimulants for Agronomic and Physiological Traits of Crops

The stimulation of germination, seedlings and plant growth as well as crop productivity in response to PBs application has been usually associated to the action of signaling bioactive molecules in the primary and secondary metabolisms (Calvo et al., 2014). Different types of hydrolyzed collagen, including granulated gelatin, gelatin hydrolysate and amino acid mixtures simulating gelatin composition, were evaluated in terms of plant growth on cucumber (Wilson et al.). In their study, the authors reported that gelatin hydrolysate treatment increased the expression of genes encoding for amino acid permeases (*AAP3*, *AAP6*) and transporters of amino acids and nitrogen. Therefore, they concluded that gelatin hydrolysate provided a sustained source of N and acted as a biostimulant. Furthermore, Luziatelli et al. conducted a greenhouse experiment on lettuce aiming to assess the effect of three commercial PBs: vegetal-derived PH, vegetal-derived PH enriched with copper and a tropical plant extract on plant growth, and the epiphytic bacterial population. The three commercial PBs boosted the shoot fresh weight with no significant differences between the three organic PBs. The authors were also able to demonstrate that PBs can stimulate the growth of epiphytic bacteria (*Pantoea*, *Pseudomonas*, *Acinetobacter*, and *Bacillus* genus) with PGP and/or biological control activity against pathogens, thus acting synergistically with organic compounds to increase marketable fresh yield of lettuce. Similarly, Mahnert et al. showed the potential of organic biostimulants (containing vermicompost, malt sprouts, stone dust, and organic herbs) to have a positive impact on plant growth and performance by shifting the microbiota on the aboveground parts of the plant as well as in the surrounding. Moreover, Lucini et al. carried out a short term experiment on melon to assess the physiological and metabolomic responses to a biopolymer-based biostimulant containing lateral root promoting peptides and lignosulphonates as well as micronutrients. The vegetal-based biostimulant was applied at four increasing concentrations (0, 0.3, 0.6, 1.2, or 2.4 L ha^-1^) 2 days after transplanting around the collar level. The substrate drench of a biopolymer-based biostimulant elicits dose-dependent (especially at 0.12 and 0.24 ml plant^-1^) increase of biomass production of melon transplants. The root trait characteristics (total root length and surface area) in biostimulant-treated plants were significantly higher at 0.24 ml plant ^-1^ and to a lesser extent at 0.12 and 0.48 ml plant^-1^, in comparison to 0.06 ml plant^-1^ and untreated melon plants. Direct and indirect physiological mechanisms were responsible for better shoot and root biomass production of treated melon transplants. For instance, the signaling molecules in particular bioactive peptides and lignosulfonates may have elicited signal transduction pathway through biosynthesis stimulation of target endogenous phytohormones (Matsumiya and Kubo, 2011). On the other hand, Palumbo et al. reported that humic acids (applied at 0.5 mg and 1 mg C L^-1^ for 2 days) extracted from olive mill water filters and municipal solid waste could be used as valuable biostimulants in maize at both concentrations as demonstrated by their capacity to promote significantly plant growth, activity of marker enzymes, and nutrient accumulation. While on maize, Ertani et al. evaluated the biostimulant effect of 6 seaweed extracts (one extract from *Laminaria* and five extracts from *Ascophyllum nodosum*) supplied for 2 days at 0.5 ml L^-1^. Thanks to a combination of morphological, chemical, and biochemical approaches, the authors demonstrated that one of the *A. nodosum* extract was the most efficient in promoting root morphological traits, likely due to its elevated content in indole-3-acetic acid. Such findings illustrate the utility of a robust chemical characterization of commercial seaweed extracts, which predicts the metabolic targets of seaweed extracts-based biostimulants before their commercialization on the market.

Additionally, a significant stimulation of plant growth parameters, yield and yield components of two greenhouse pepper cultivars was observed when seedlings were exposed to *Cladosporium sphaerospermum* (Li et al.). Result of the same study showed that tobacco plants exposed to *C. sphaerospermum* retained higher rates of growth, where it was associated with several putative physiological and molecular mechanisms including cell expansion and cycle, photosynthesis, phytohormone homeostasis, and defense responses.

Concerning flower crops, Cristiano et al. investigated the application effect of an animal-based PH as foliar spray or as substrate drench, applied at three doses (0, 0.1, and 0.2 g L^-1^) on the agronomical and physiological responses of two snapdragon hybrids. At both PB doses, the application of animal-based PH especially as substrate drench enhanced the performance parameters and ornamental quality traits of snapdragon in a cultivar-dependent manner, compared to untreated control treatment.

In addition to the stimulation action of microbial and non-microbial PBs, the application of these natural substances or microorganisms can have a dual effect including tolerance to both biotic and abiotic stressors. For instance, Sharma et al. study, showed that the exogenous application of jasmonic acid can aid *Brassica juncea* seedlings in recovering from the negative impact of oxidative stress caused by pesticide toxicity, throughout the up-regulation of *RUBISCO*, *NADH*, *CXE*, and *P450* and by triggering the antioxidative defense system of the plants. Similarly, *Trichoderma erinaceum* bio-priming modulated tomato defense transcriptome after the challenged conditions of *Fusarium oxysporum* f. sp. *lycopersici*, where the plants were accompanied by (i) improved accumulation of defense-related WRKY (a class of DNA-binding proteins) transcripts, (ii) increased antioxidative enzyme activities, and (iii) reinforced through a higher number of lignified cell layers, leading to a higher plant growth (Aamir et al.). Finally, Dal Cortivo et al. showed that sedaxane, a succinate dehydrogenase inhibitor with a well know fungicide action, exhibited also a significant hormone-like activity (i.e, auxin-like and gibberellin-like effects) when applied to maize seeds. The authors concluded that sedaxane application can facilitate root establishment and intensify N and phenylpropanoid metabolism in young maize, thus overcoming both biotic and abiotic pressure in early growth stages.

## Implications of Biostimulants for Abiotic Stresses Tolerance

Unfavorable environmental and soil conditions in particular drought, salinity, and extreme temperature are responsible for 70% of yield gap dictated by global climatic changes (Wang et al., 2003). According to the actual climate change scenario, these abiotic stresses are expected to have an increased negative impact, posing serious concerns on crop productivity, and thus food security worldwide (Rouphael et al., 2018b). In order to overcome this situation, the application of non-microbial and microbial PBs has been suggested as one of the most promising and efficient drivers toward further yield stability (Rouphael et al., 2018a).

The application of a legume-based PH (containing amino acids and soluble peptides), as foliar and especially as drench substrate, was found to mitigate the negative effects of drought in tomato grown in controlled environment, by increasing transpiration use efficiency (Paul et al.). The metabolomic approach adopted in this study allowed the identification of the molecular mechanisms of improved drought tolerance following the biostimulant treatment, such as (i) improved tolerance to ROS-mediated (ii) modulation of phytohormones and lipids profiles. The hormonal effects of an animal-based PH (containing L-α amino acids, free amino acids, organic-nitrogen, iron, and potassium) on water-stressed tomato plants were also assessed by a Spanish group (Casadesus et al.). Results of the greenhouse experiment showed that the application of animal-based PH benefited an antioxidant protection and exerted a major hormonal effect in tomato water-stressed leaves by increasing the endogenous content of auxin, cytokinin, and jasmonic acid. Microbial biostimulants based on AMF were also reported to promote tolerance of tomato plants toward drought stress. In the study of Volpe et al., the impact of two AMF strains *Funneliformis mosseae* and *Rhizophagus intraradices* on physiological and molecular responses of tomato were evaluated. The contribution of *F. mosseae* seems more effective on volatile organic compounds production, whereas *R. intraradices* exhibited the best performance traits, leading to a significant higher water use efficiency under severe drought stress. Additionally, *R. intraradices* was demonstrated to be effective against combined abiotic and biotic stress, the latter in terms of attraction toward aphids natural enemies. Moreover, in tomato cultures Bitterlich et al. showed that mycorrhizal plants indeed show higher water extraction rates per unit root length and biomass which was a consequence of AMF-mediated substrate hydraulic properties. The alleviation of substrate water flow resistances in AMF pots allowed for higher root extraction rates and maintenance of transpiration under progressive drought when the potential soil water flow to root systems were limiting transpiration rates (Bitterlich et al.). Because this study indicated that enhanced water extraction capacity in mycorrhizal pots was related to the flow of water from the bulk substrates to the root surface, the same group of authors carried out an additional study in order to see whether AMF substrate colonization under root exclusion is sufficient to alter substrate hydraulic properties (Bitterlich et al.). Indeed, substrate colonization by AMF that engaged in a functional symbiosis stabilized water retention and enhanced unsaturated hydraulic conductivity of the substrate. Theoretically, enhanced hydraulic conductivity in AMF substrates constitutes an effective enlargement of the water depletion zone around roots. The authors concluded that further studies should investigate how this would quantitatively contribute to water acquisition by plants and the variability of the effect across different soils.

Characterization of several halotolerant PGPR (*Bacillus* spp.) isolated from the rhizosphere of durum wheat cultivated in hypersaline environments, revealed several growth promoting traits (Verma et al.). Several combinations of these PGPR strains were able to boost plant growth traits of mungbean. The authors concluded that specific strains such as *Bacillus* sp. BHUJP-H1 and *Bacillus* sp. BHUJP-H2 can be used as drought tolerant PGPR under open field conditions.

Non-microbial and microbial PBs can be also considered a possible way to enhance tolerance to salinity. Zou et al. reported that the application of crude polysaccharides from brown seaweed *Lessonia nigrescens* or the application of separated and fractionated acidic polysaccharides: LNP-1 at 40.2 kDa and especially LNP-2 at 63.9 kDa, improved the salinity tolerance of wheat seedlings. These beneficial effects were associated to several biochemical and physiological mechanisms such as (i) decreased membrane lipid peroxidation, (ii) increased chlorophyll content, (iii) improved antioxidant activities, and (iv) a better efflux and compartmentation of intracellular ion. The same group of authors, also demonstrated that not only polysaccharides deriving from brown algae but also those deriving from red algae (*Pyropia yezoensis*) can mitigate the negative effects of salinity on wheat seedlings grown under saline conditions (Zou et al.). In their second study, polysaccharides with different molecular weights (3.2, 10.5, 29.0, and 48.8 kDa) were prepared. The authors concluded that the lower-molecular weight samples (3.2 kDa) protected most effectively wheat seedlings against salt stress damage, by coordinating the efflux and compartmentation of NaCl and by enhancing antioxidant activities (Zou et al.). The use of a biostimulant product based on carboxylic acids, containing calcium oxide complexed by ammonium ligninsulfonate was tested on greenhouse lettuce, and it was proven to improve tolerance to nutrient solutions of high electrical conductivity (Bulgari et al.). Lettuce plants treated especially at the higher dose (0.2 ml/L), showed a significant increase in fresh biomass, which was associated to a better biochemical and physiological status (higher chlorophyll content and net photosynthetic rate). Similarly, Wu et al. demonstrated that exogenous 5-aminolevulinic acid application minimized NaCl toxicity on cucumber seedlings through improvement in chlorophyll synthesis, light harvesting capacity, photosynthesis capacity and retarded thylakoid degradation. Moreover, the beneficial role of small bioactive molecules (< 500 Da) such as omeprazole (OMP) a benzimidazole inhibitor of animal proton pumps was reported by Rouphael et al. Salt-stressed tomato plants treated with 10 or 100 μM OMP as substrate drench modulated root system architecture in terms of total length and surface, leading to a higher nutrient uptake and biomass production. Hormonal network was strongly influenced by OMP, eliciting an increase in ABA, a decrease in auxins and cytokinin, as well as a tendency in GA down accumulation. Finally, Albdaiwi et al. selected several potential bacterial isolates possessing plant growth promoting traits including N fixation, auxin and siderophore production and inorganic phosphate solubilization. The authors showed that six halotolerant PGPR strains were able to enhance survival in inoculated plants under high salt stress conditions as reflected by higher agronomic performance (higher germination percentages and seedling root growth) of durum wheat in comparison with non-inoculated plants.

## Implications of Biostimulants for Improving Nutrient Use Efficiency

The use of bioactive natural substances and microbial inoculants can represent a valuable tool to enhance soil nutrient availability, plant nutrient uptake and assimilation (De Pascale et al., 2017). Increasing nutrient use efficiency in particular N and P is fundamental for both economical and environmental reasons. At both optimal and sub-optimal N regimens (112 and 7 mg L^-1^, respectively) the application of legume-derived PH especially as substrate drench improved leaf number, SPAD (Soil Plant Analysis Development) index, and biomass production of greenhouse tomato (Sestilli et al.). The better agronomic responses of PH-treated tomato was associated to the stimulation of root apparatus that facilitated N uptake and translocation. Moreover, under sub-optimal N concentrations, PH application upregulated the expression of genes encoding for amino acid transporter and ferredoxin-glutamate synthases and glutamine synthetase in roots, which are known to be involved in N assimilation. Furthermore, the biostimulant action of two strains of *Trichoderma* (*T. virens* GV41 or *T. harzianum* T22), under suboptimal, optimal, and supraoptimal levels of N in two leafy vegetables: lettuce and rocket was investigated by Fiorentino et al. The authors reported that *T. virens* GV41 improved Nitrogen Use Efficiency (NUE) of lettuce, and favored the uptake of native N present in the soil of both leafy vegetables. The beneficial effect of microbial-based biostimulants was species-dependent with more pronounced effects recorded on lettuce. The findings also demonstrated that *Trichoderma* inoculation strongly modulated the composition of eukaryotic populations in the rhizosphere, by exerting different effects with suboptimal N regimen compared to N fertilized treatments. In addition to beneficial fungi, bacterial inoculants could also improve the availability of nutrients and their utilization by plants. In Koskey et al. work, 41 rhizobia isolates from root nodules of mild altitudes climbing bean varieties were characterized from a morpho-cultural, biochemical, and genetic point of view in order to select strains with potential biofertilizer properties able to perform under diverse environments. The use of multiple microbial inoculants (bacteria + fungi) containing *Agrobacterium*, *Azotobacter*, *Azospirillum*, *Bacillus*, *Pseudomonas*, *Streptomyces*, *Trichoderma*, and *R. irregularis* was found effective for wheat production compared to the commercial mineral and chemical fertilizers applied at the recommended level for on-farm use in south-western Australia characterized by moderately N and P deficient soil (Assainar et al.). Zinc solubilization by PGPR is relatively a newer approach, thus a research group from Pakistan screened zinc solubilizing rhizobacteria isolated from wheat and sugarcane and analyzed their effects on wheat (Kamran et al.). The authors reported the potential of *Pantoea*, *Enterobacter cloacae*, and especially *Pseudomonas fragi* to be used as microbial-based biostimulant to overcome zinc deficiency under low input scenario.

## Implications of Biostimulants for Enhancing Produce Quality

The application of microbial and non-microbial plant biostimulants are able to modify plant primary and secondary metabolism (Colla et al., 2015; Rouphael et al., 2015) leading to the synthesis and accumulation of antioxidant molecules (i.e., secondary metabolites) which are important for human diet. The application of earthworm grazed and *Trichoderma harzianum* biofortified spent mushroom substrate (SMS) induced a significant increase in tomato fruit quality in terms of antioxidant capacity, total soluble sugars, carotenoids (lycopene, lutein, and β-carotene), total polyphenols, and flavonoids contents as well as mineral composition (P, K, Ca, Mg, Fe, Mn, and Zn) (Singh et al.). Moreover, Trejo-Téllez et al. investigated the effect of photosynthetically active radiation (low or high), phosphate (low or high), and phosphite (low, optimal or high), and their interactions on the concentrations of glucosinolates, flavonoids, and nitrate in two *Brassica* species: *Brassica campestris* and *Brassica juncea*. The authors reported that the application of phosphite in the nutrient solution tends to increase phosphate deficiency; therefore, it favors the biosynthesis and accumulation of some target flavonoids and glucosinolates as a possible defense mechanism for coping with nutrient stress.

Concerning fruit trees and grapevines, several authors (Soppelsa et al.; D'Amato et al.; Vergara et al.; Koyama et al.) investigated the application of PBs or exogenous molecules on nutritional and functional quality of fruits. Biostimulant products based on *A. nodosum* seaweed extract, PH, and B-group vitamins had a minor impact on primary apple quality traits (size, flesh firmness, acidity, and total sugars), whereas they induced an improvement of the intensity and extension of red coloration in “Jonathan” apples at harvest in the 2 years trials (Soppelsa et al.). Moreover, the foliar application of Se on olive trees improved nutritional and functional qualities of Extra Virgin Olive Oil (EVOO); since besides the Se biofortification effect, an accumulation of antioxidants molecules was recorded by D'Amato et al. In their study, the biosynthesis and accumulation of key antioxidant molecules such as carotenoids and phenols may have brought advantages to EVOO itself, by improving its oxidative stability and consequently its shelf-life.

In “Redglobe” table grape, treatments with 3 brassinosteroids analogs (24-epibrassinolide, Triol, or Lactone at three concentrations 0.0, 0.4, or 0.8 mg L^-1^) or a commercial formulation (B-2000R at 0.06 mg L^-1^) at the onset of véraison, improved total soluble solids, berries color, and anthocyanins without altering yield (Vergara et al.). In line with the previous study, the exogenous application of abscisic acid at different timings (7 or 21 days after véraison; DAV) and concentrations (200 or 400 mg L^-1^) modulated the biosynthesis of anthocyanins and flavonoids in *Vitis vinifera* × *Vitis labrusca* table grapes (Koyama et al.). The authors showed that two applications (at 7 and 21 DAV) of abscisic acid at 400 mg L^-1^, resulted in an increase in (i) concentrations of the total and individual anthocyanin, (ii) expression of the key biosynthetic genes *CHI*, *DFR*, *F3H*, and *UFGT*, and (iii) expression of the transcription factors *VvMYBA1* and *VvMYBA2*.

## Outlook and Challenges Ahead

PBs including natural substances and microbial inoculants appear as a novel and potential category of agricultural inputs, complementing agrochemicals including synthetic fertilizers, and improving tolerance to abiotic stresses, as well as enhancing the quality of agricultural and horticultural commodities. Characterizing the bioactive components of PBs and elucidating the molecular and physiological stimulation mechanisms are still of high interest for the scientific community and commercial enterprises. Due to the complex matrices with different groups of bioactive and signaling molecules, the use of small/medium/large high-throughput phenotyping is the most efficient technology to develop novel biostimulants (Rouphael et al.). Ugena et al. demonstrated that multi-trait high-throughput screening is suitable for identifying new potential biostimulants and characterizing their mode of action under both optimal and sub-optimal (i.e., salinity) conditions. Based on this novel technology, the authors concluded that the mode of action of PBs could be summarized in three groups: (i) plant growth promotors/inhibitors, (ii) stress alleviators, and (iii) combined action. Similarly, Paul et al. reported that the combined use of high-throughput phenotyping and metabolomics could facilitate the screening of new bioactive and signaling substances with biostimulant properties and could provide a biochemical, morpho-physiological, and metabolomic gateway to the mode of actions, underlying PHs action on tomato. Finally, Rouphael and Colla suggested that in the near future the main players of PBs (scientists, private industries, legislators, and stakeholders) should focus on the development of a second generation of these products (biostimulant 2.0) with specific synergistic biostimulatory action through the application of both microbial and non-microbial PBs to render agriculture more sustainable and resilient.

## Author Contributions

YR and GC have made a substantial, direct, and intellectual contribution to the work, and approved it for publication in Frontiers in Plant Science.

## Conflict of Interest

The authors declare that the research was conducted in the absence of any commercial or financial relationships that could be construed as a potential conflict of interest.
